# Effects of arachidonic acid supplementation on training adaptations in resistance-trained males

**DOI:** 10.1186/1550-2783-4-21

**Published:** 2007-11-28

**Authors:** Michael D Roberts, Mike Iosia, Chad M Kerksick, Lem W Taylor, Bill Campbell, Colin D Wilborn, Travis Harvey, Matthew Cooke, Chris Rasmussen, Mike Greenwood, Ronald Wilson, Jean Jitomir, Darryn Willoughby, Richard B Kreider

**Affiliations:** 1 Department of Health and Exercise Science, University of Oklahoma, Norman, OK, USA; 2 Department of Health, Exercise Science and Secondary Education, Lee University, Cleveland, TN, USA; 3 Department of Health, Human Performance & Recreation, Baylor University, Waco, TX, USA; 4 Department of Health, Leisure, and Exercise Science, University of West Florida, Pensacola, FL, USA; 5 School of Physical Education and Exercise Science, University of South Florida, Tampa, FL, USA; 6 Department of Exercise and Sport Science, University of Mary Hardin-Baylor, Belton, TX, USA; 7 Department of Physical Education, United States Military Academy, West Point, NY, USA

## Abstract

**Background:**

To determine the impact of AA supplementation during resistance training on body composition, training adaptations, and markers of muscle hypertrophy in resistance-trained males.

**Methods:**

In a randomized and double blind manner, 31 resistance-trained male subjects (22.1 ± 5.0 years, 180 ± 0.1 cm, 86.1 ± 13.0 kg, 18.1 ± 6.4% body fat) ingested either a placebo (PLA: 1 g·day^-1 ^corn oil, n = 16) or AA (AA: 1 g·day^-1 ^AA, n = 15) while participating in a standardized 4 day·week^-1 ^resistance training regimen. Fasting blood samples, body composition, bench press one-repetition maximum (1RM), leg press 1RM and Wingate anaerobic capacity sprint tests were completed after 0, 25, and 50 days of supplementation. Percutaneous muscle biopsies were taken from the vastus lateralis on days 0 and 50.

**Results:**

Wingate relative peak power was significantly greater after 50 days of supplementation while the inflammatory cytokine IL-6 was significantly lower after 25 days of supplementation in the AA group. PGE_2 _levels tended to be greater in the AA group. However, no statistically significant differences were observed between groups in body composition, strength, anabolic and catabolic hormones, or markers of muscle hypertrophy (i.e. total protein content or MHC type I, IIa, and IIx protein content) and other intramuscular markers (i.e. FP and EP_3 _receptor density or MHC type I, IIa, and IIx mRNA expression).

**Conclusion:**

AA supplementation during resistance-training may enhance anaerobic capacity and lessen the inflammatory response to training. However, AA supplementation did not promote statistically greater gains in strength, muscle mass, or influence markers of muscle hypertrophy.

## Background

Nutritional supplements are often marketed to resistance-trained athletes as nutritional ergogenic aids purported to promote gains in strength, muscle mass, and/or performance during training. For example, creatine, amino acids (i.e., branched chain amino acids, arginine, etc.), polyunsaturated fatty acids (i.e., fish oils), dihydroepiandrosterone (DHEA), and antioxidants (i.e. vitamin E, catechins, etc.) have been shown to exhibit anabolic, anti-catabolic, and/or ergogenic properties in young, healthy [1] and diseased populations [2-6]. Consequently, although equivocal data exists [7,8], supplements have also been marketed as ergogenic aids for athletes. Moreover, new nutrients are continually introduced as potential nutritional ergogenic aids for this population. While there is some basic and/or applied data available to support the potential ergogenic value of some nutritional supplements, many lack data supporting efficacy and/or safety [9].

A nutrient that has recently been purported to possess anabolic properties and marketed to resistance-trained athletes is arachidonic acid (AA). Arachidonic acid (20:4, ω-6) is a polyunsaturated fatty acid that is consumed in low amounts in the diet and is relatively abundant in membrane phospholipids. The fatty acid content in sarcoplasmic membrane phospholipids is thought to be contingent upon dietary habits [10,11] and physical activity levels [12,13]. Consequently, a high dietary intake of ω-6 fatty acids increases the endogenous ω-6 fatty acid content of membrane phospholipids, while active individuals present a lower ω-6-to-ω-3 ratio compared to their sedentary counterparts, respectively. Furthermore, AA has been reported to be a bioactive compound involved in myogenic inflammation processes that occurs in response to mechanical strain such as resistance-training [14,15]. In this regard, products of AA metabolism include explicit prostaglandin isomers (i.e., PGE_2_, PGF_2α_), which are formed through the cyclooxygenase-2 isozyme (COX-2) pathway. Recent studies examining the inhibition of the COX-2 pathway have discerned that COX-2 isozyme products mitigate protein degradation and subsequent myofibrillar regeneration in skeletal muscle [16,17]. A recent investigation by Trappe *et al. *[18] demonstrated that when males supplemented with COX-2 inhibitors (i.e., ibuprofen and acetaminophen) prior to resistance training, post-exercise PGF_2α _production and muscle protein synthesis was completely abolished following an eccentric resistance training protocol. Trappe and colleagues concluded that COX-2 inhibition inhibited post-exercise protein synthesis due to the cessation of PGF_2α _formation [18]. In addition, previous research suggests that 1.5 g·d^-1 ^of AA supplementation for 50 d in young, healthy men significantly increased leukocytic prostaglandin production [19], although, no measures of protein synthesis were made in this study.

Therefore, it is plausible that AA levels are lower in resistance-trained individuals due to their elevated activity levels, and that supplementation may increase intramuscular AA pools during resistance training. Due to the fact that AA is a substrate of the COX-2 isozyme that is converted into PGF_2α_, potentially increasing intracellular AA via nutritional supplementation may potentiate the post-exercise production of PGF_2α_. Furthermore, possible post-exercise increases in PGF_2α _with AA supplementation may further enhance muscle protein synthesis and lead to subsequent muscle hypertrophy over chronic supplementation periods with concurrent resistance training. However, we are aware of no research that has examined the effects that AA supplementation on circulating prostaglandins and/or skeletal muscle mass and function. Therefore, the purpose of this study was to investigate whether AA supplementation in conjunction with resistance training affects body composition, training adaptations, and hormonal and cellular markers of muscle hypertrophy and inflammation in resistance-trained individuals.

## Methods

### Subjects

Thirty-one healthy, resistance trained males (22.1 ± 5.0 yrs, 180 ± 0.1 cm, 86.1 ± 13.0 kg, 18.1 ± 6.4% body fat) were informed of study protocol approved by the Institutional Review Board at Baylor University prior to participation. Training status was self-reported, and individuals who lacked at least one year of experience prior to study were excluded. In addition, subjects were excluded if they: 1) had any history of metabolic, hypertension, hepatorenal, musculoskeletal, autoimmune, or neurologic disease; 2) were currently taking thyroid, antihyperlipidemic, hypoglycemic, anti-hypertensive, or androgenic medications; and 3) had taken nutritional supplements that may affect muscle mass [i.e., creatine, hydroxy-beta-methylbutyrate (HMB)] and/or anabolic/catabolic hormone levels [i.e. androstenedione, dihydroepiandrosterone (DHEA), or other prohormones] within three months of the starting the study.

### Baseline testing

Eligible subjects were familiarized to the study protocol via a verbal and written explanation of the study design. Subjects signed an informed consent statement and completed personal and medical histories while also completing a Wingate anaerobic capacity test prior to baseline testing. Subjects were instructed to refrain from strenuous exercise for 48 hours and fast for 10 hours prior to baseline testing (i.e., day 0) which occurred 3–4 days following familiarization to allow for recording of dietary intake. This study employed a double-blind, placebo-controlled, parallel study design whereby subjects were matched evenly into clusters according to age and body mass.

### Experimental protocol

During days 0, 25, and 50 each subject reported to the laboratory after a 10-hour fast. Height was measured using standard anthropometry and total body mass was measured using a calibrated Healthometer digital strain gauge electronic scale (Bridgeview, IL) with a precision of ± 0.02 kg. Body composition was then determined using a calibrated Hologic Discovery W dual-energy x-ray absorptiometer (DXA) device (Hologic Inc., Bedford, MA), while blood pressure and resting heart rate was determined using standard procedures. Subjects then donated approximately 25 ml of fasting blood using standard venipuncture techniques for hematological, clinical chemistry panels and later cytokine and hormone analysis. Two 10 ml serum separation vacutainer tubes and one 5 ml K_3 _EDTA vacutainer tube were inserted into the vacutainer holder for blood collection in succession using multiple sample phlebotomy techniques. Whole blood was immediately analyzed for a complete blood count while serum vacutainer tubes were centrifuged at room temperature for 15 min at 1,500 *g*, the serum supernatant was transferred into microcentrifuge tubes, and the serum samples were stored at -20°C for subsequent hormonal and metabolite analyses. On days 0 and 50 only, subjects donated approximately 60 mg of skeletal muscle from the vastus lateralis using the Bergstrom biopsy technique. Upper and lower body strength were assessed using standard 1RM testing procedures [20] with a bench press and 35° hip sled machine (Nebula, Versailles, OH). Test-retest reliability of these strength tests on resistance-trained subjects in our laboratory have yielded a high reliability for the bench press (*r *= 0.94) and leg press (*r *= 0.91). After determination of hip sled 1RM, subjects rested 10 minutes before completing a 30 seconds Wingate anaerobic capacity test on a computerized cycle ergometer (Lode Excalibur, Lode, Groningen, The Netherlands) to assess lower body anaerobic power. This test consisted of having each subject sprint in an all out fashion on the bicycle ergometer for 30 seconds against a standard workload of 0.075 kg·kg^-1 ^of body weight. Test-retest reliability for absolute peak power and mean power in our laboratory have also yielded high reliability values (*r *= 0.69 and *r *= 0.95, respectively, *P *< 0.05).

### Percutaneous muscle biopsies

Muscle biopsies were taken on days 0 and 50 prior to all strength testing to avoid potential myofibrillar disruption due to exercise [21]. Subjects were instructed to refrain from exercise 48 hours prior to each muscle biopsy. Muscle was extracted from the lateral portion of the *vastus lateralis *midway between the patella and iliac crest of the dominant leg using a 5 mm biopsy needle with applied suction [22]. Briefly, 1.5 ml of 1.0% Lidocaine HCl was injected subcutaneously prior to making a small pilot incision. Using double-chop procedures and applied suction, the specimen first had all visible fat and connective tissue removed prior to being flash frozen in liquid nitrogen. All samples were subsequently stored at -80°C until later analyses.

### Supplementation protocol and dietary monitoring

In a double-blind fashion, subjects ingested four 250 mg capsules containing a corn oil placebo or AA (X-Factor, Molecular Nutrition, Jupiter, FL) over 50 days following baseline testing. Supplements were prepared in capsule form and packaged in generic bottles by Molecular Nutrition. Compliance was monitored by having subjects return empty supplement bottles after 25 and 50 days of supplementation. In accordance with previous guidelines and in an effort to ensure energy and protein intake were adequate to facilitate muscle hypertrophy, all subjects were instructed to increase caloric intake by approximately 500 kcal·day^-1 ^while also maintaining an estimated protein intake of 2 g·kg^-1^·day^-1 ^when compared to baseline dietary analysis [23]. Subjects were provided a commercially-available meal replacement powder (Lean Body, Labrada Nutrition, Houston, TX) containing approximately 290 kilocalories, 24 g of carbohydrate, 45 g of protein and 1 g of fat per serving in an attempt to accommodate the above mentioned energy and protein requirements. Depending on baseline protein intake, subjects were told to ingest 1 to 2 packets of the meal replacement supplement in the morning and/or immediately following each workout [24]. Additionally, subjects were instructed to avoid regular consumption of foods known to be high in ω-3 fatty acids including fish oil, flaxseed oil, cold water fish, olive oil, sesame oil, peanut butter, N-acetyl-cysteine, conjugated linoleic acid, as well as anti-inflammatory medications including acetaminophen, ibuprofen, aspirin and other non-steroidal anti-inflammatory drugs [18]. Dietary intake as well as linoleic (18:2, ω-6), linolenic (18:3, ω-3), and AA intake were monitored with 4-day dietary recalls at days 0, 25 and 50 and assessed using the Food Processor III Nutrition Software (ESHA Nutrition Research, Salem, OR).

### Resistance-training protocol

Over a 50-day period, subjects completed a 4 day·week^-1 ^split-body, linear periodization resistance-training program. Upper body lifts included bench press, lat pull, shoulder press, seated rows, shoulder shrugs, chest flies, biceps curls, and triceps press-downs while lower body lifts included leg press, back extension, step ups, leg curls, leg extension, heel raises, and abdominal crunches twice per week. Subjects performed 3 sets of 10 repetitions with as much weight as they could lift per set (i.e., 60–80% of 1RM). Rest periods between exercises did not exceed 3 minutes, while the rest between sets did not exceed 2 minutes. Training was conducted at the university's student life center, documented in training logs, and signed off by designated staff members to verify compliance and monitor progress. This protocol has been shown in prior research to promote significant gains in muscular strength, muscular endurance, and fat free mass [25].

### Serum and whole blood analyses

Serum and whole blood samples were used to evaluate clinical safety during the supplementation protocols. Serum samples were assayed for comprehensive metabolic panels including glucose, total protein, blood urea nitrogen (BUN), creatinine, BUN/creatinine ratio, uric acid, aspartate aminotransferase (AST), alanine aminotransferase (ALT), creatine kinase (CK), lactate dehydrogenase (LDH), gamma-glutamyl transpeptidase (GGT), albumin, globulin, sodium, chloride, calcium, carbon dioxide, total bilirubin, alkaline phosphatase (ALP), triglycerides, cholesterol, HDL and LDL using a Dade Dimension XL clinical chemistry system (Dade Behring Inc., Newark, DE). Complete blood cell counts including red cell counts, hemoglobin, hematocrit, mean cell volume, mean corpuscular hemoglobin, mean corpuscular hemoglobin concentration, red cell distribution width, white blood cell counts, neutrophils, lymphocytes, monocytes, eosinophils, and basophils were analyzed via flow cytometry using the Cell-DYN 1800 (Abbott Laboratories, Abbott Park, IL). Test to test reliability (within and between) of performing these assays ranged from 2 to 6% for individual assays with an average coefficient of variation (C_*V*_) of 3.0%. Samples were run in duplicate to verify results if the observed values were outside control values and/or clinical norms according to standard procedures.

Subsequent serum samples were later assayed for cortisol (CORT), free testosterone (fTEST), total testosterone (tTEST), interleukin-6 (IL-6), prostaglandin E_2 _(PGE_2_) and prostaglandin F_2α _(PGF_2α_). Commerical enzyme immunoabsorbent assays were used to analyze serum concentrations of PGF_2α_, PGE_2_, and IL-6 (Cayman Chemical, Ann Arbor, MI) and CORT, fTEST, and tTEST (Diagnostic Systems Laboratories, Webster, TX). The C_*V *_of performing these EIA-based assays ranged from 3.0 to 5.0%.

### Total RNA isolation

Total cellular RNA was extracted from the homogenate of biopsy samples with a monophasic solution of phenol and guanidine isothiocyanate contained within the TRI-reagent (Sigma Chemical Co., St. Louis, MO) [26-30]. Total RNA concentrations from each sample were determined spectrophotometrically with an optical density of 260 nm (OD_260_), with final concentration adjusted to 200 ng·μl^-1 ^by diluting the crude total RNA extracts into DEPC-treated nuclease-free H_2_O. This procedure has been shown to yield un-degraded RNA, free of DNA and proteins as indicated by prominent 28S and 18S ribosomal RNA bands, as well as an OD_260_/OD_280 _ratio of approximately 2.0 [26-30]. The RNA samples were stored at -80°C until later analyses.

### Reverse transcription and clonal DNA synthesis

The standardized solutions of total cellular RNA were reverse transcribed to synthesize clonal DNA (cDNA) as described previously [26-30]. In short, a reverse transcription reaction mixture [i.e., 1 μl of total cellular RNA, 4 μl 5× reverse transcription buffer, a dNTP mixture containing dATP, dCTP, dGTP, and dTTP, MgCl_2_, RNase inhibitor, an oligo(dT)_15 _primer, 10 μL of nuclease-free H_2_O and 1 U·μl^-1 ^MMLV reverse transcriptase enzyme (Bio-Rad, Hercules, CA)] were incubated at 42°C for 40 minutes, heated to 85°C for 5 minutes, and then quick-chilled on ice yielding the cDNA product. Starting cDNA template concentrations were standardized to 200 ng·μl^-1 ^prior to real-time polymerase chain reaction (RT-PCR) amplification by detecting crude cDNA synthesized products spectrophotometrically at a wavelength of 260 nm and diluting them in nuclease-free H_2_O. The standardized cDNA solutions were frozen at -80°C until real-time RT-PCR was performed.

### RT-PCR

Anti-sense and sense oligonucleotide primer pairs were constructed using commercially available Beacon Designer software (Bio-Rad, Hercules, CA) from known mRNA sequences published in the GenBank nucleotide database [31] and commercially synthesized (Integrated DNA Technologies, Coralville, IA). The following 5' sense and 3' anti-sense oligonucleotide primers were used to isolate the three adult MHC isoforms (Type I, IIa, and IIx): Type I MHC mRNA (5' primer: bases 776–796, 3' primer: bases 1398-1378, GenEMBL AC X06976), Type IIa MHC mRNA (5' primer: bases 1785–1805, 3' primer: bases 2440-2420, GenEMBL AC AF111784), Type IIx MHC mRNA (5' primer: bases 1138–1158, 3' primer: bases 1746-1726, GenEMBL AC AF111785). These primers amplify fragments of 141, 145, and 148 base pairs, respectively, for Type I, IIa, IIx MHC. β-actin was used as an external reference standard for detecting relative change in the quantity of target mRNA due to its consideration as a constitutively expressed housekeeping gene [32], These β-actin primers amplify a PCR fragment of 135 base pairs. Two hundred ng of cDNA was added to each of the four PCR reactions for MHC Type I, -IIa, and -IIx, and β-actin. Specifically, each PCR reaction contained the following mixtures: 2 μl of cDNA template was added along with 12.5 μl of 2× SYBR Green Supermix (Bio-Rad, Hercules, CA) [100 mM KCl mixture, 40 mM Tris-HCl, 0.4 mM of each dNTP, 50 U·ml^-1 ^of iTaq DNA polymerase, 6.0 mM MgCl_2_, SYBR Green I, 20 nM flourescein], 1.5 μl of sense and anti-sense primers and 7.5 μl nuclease-free dH_2_O]. Each PCR reaction was amplified with a thermal cycler (Bio Rad, Hercules, CA) and the amplification sequence involved a denaturation step at 95°C for 30 seconds, primer annealing at 55°C for 30 seconds, and extension at 72°C for 60 seconds [27,33,34]. RT-PCR was performed over 40 cycles with emitted fluorescence from the SYBR green fluorophore being measured after each cycle. An emission of fluorescence occurs due to the integration of the SYBR green into the double-stranded cDNA produced during the PCR reaction. All C_T _values were assessed in the linear portion of amplification and a DNA melting curve analysis was performed after amplification to assure that the single gene products were amplified in absence of primer-dimers. Quantification of all mRNA was expressed relative to β-actin expression. A comparison of C_T _value ratios [Day 0 (MHC mRNA C_T_/β-actin mRNA C_T_) versus Day 50 (MHC mRNA C_T_/β-actin mRNA C_T_)] were used to compare changes in basal gene expression between the AA and PLA groups. Agarose gel electrophoresis using 25 μl aliquots of the finalized PCR reaction mixtures was performed in 1.5% agarose gels [1 μg·ml^-1^] using 1× Tris-Boric acid-EDTA (TBE) buffer and illuminated with a UV transilluminator (Chemi-Doc XRS, Bio-Rad, Hercules, CA) to verify positive amplification of target mRNA (data not shown) [33,34]. The C_*V *_for MHC I, IIa, and IIx were 2.06%, 3.18%, and 2.73%, respectively [33].

### Total muscle protein quantitation

Total protein remaining from the total RNA isolation procedure was isolated with isopropanol, ethanol, and 0.3 M guanidine hydrochloride. Myofibrillar protein was isolated with 0.1% sodium dodecyl sulfate (SDS) [35], prior to having protein content determined spectrophotometrically using a Bradford assay at a wavelength of 595 nm. A standard curve was generated using (r^2 ^= 0.98, *P *< 0.001) bovine serum albumin as the standard and represented relative to muscle wet weight [36]. Each protein sample was subsequently diluted to 50 μg of protein per 30 μl SDS buffer for subsequent immunoblotting. The C_*V *_for myofibrillar protein was 2.03% [28].

### MHC protein isoform quantitation

The composition of MHC protein isoforms within each muscle homogenate sample was determined by automated SDS-PAGE using Experion Pro260 chips (Bio-Rad, Hercules, CA). Approximately 6 μl aliquots of each sample were pipetted into each sample well on the microchip. Each unknown sample was prepared from 4 μl of the protein dilution from each subject (or 4 μl of the molecular weight ladder), 2 μl of sample buffer with β-mercaptoethanol, and 84 μl of de-ionized water. Based upon the findings of Gazith and colleagues [37], all three MHC isoforms were expected to migrate in the 200–210 kiloDaulton region within the polyacrylamide gel relative to the molecular weight ladder. The gels were digitally visualized by the Experion software (Bio-Rad, Hercules, CA) and MHC concentrations in each sample were assessed by comparing the arbitrary density of each MHC isoform to the arbitrary densities of molecular weight markers with known concentrations. The C_*V *_of protein bands ≥10 kD were ≤1.1%.

### Protein immunoblotting

PGF_2α _(FP) and PGE_2 _(EP_3_) receptor quantitation was performed at room temperature by extracting total muscle protein from the homogenate and slot-blotting 50 μg of total protein onto nitrocellulose membranes using a Bio-Dot protein blotting system (Bio-Rad, Hercules, CA). The blotted membranes were incubated with blocking solution for 1 hour on an orbital rocker, decanted and membranes were incubated with a TTBS wash solution for 5 minutes for a total of three washes. The membranes were incubated with specific anti-FP receptor and anti-EP_3 _receptor polyclonal antibodies (Cayman Chemical, Ann Arbor, MI), diluted to 4 μg·ml^-1^, for 1 to 2 hours on an orbital rocker. Primary antibody solutions were then decanted and the membranes washed with TTBS solution for 5 minutes on an orbital rocker for a total of three washes. The TTBS wash solution was decanted and the membranes were incubated with a secondary biotinylated goat anti-rabbit antibody solution (Bio-Rad, Hercules, CA) for 1 hour on an orbital rocker. Continuing, the secondary biotinylated goat anti-rabbit antibody solution was decanted and the membranes incubated in TTBS wash solution for a total of three washes at 5 minutes per wash. The membranes were incubated with a streptavidin-biotinylated alkaline phosphatase complex solution (Bio-Rad, Hercules, CA) for 1 hour on an orbital rocker. Finally, the streptavidin-biotinylated alkaline phosphatase complex solution was decanted and the membranes washed three times with TTBS solution at 5 minutes per wash on an orbital shaker. Color development solution containing BCIP/NBT (Bio-Rad, Hercules, CA) was added and color development was monitored over 30 – 60 minutes. The color development was stopped by incubating the membrane in double distilled H_2_O for 10 minutes on an orbital rocker. Blotted membranes were digitized by way of densitometry using a Chemi-Doc XRS imaging system (Bio-Rad, Hercules, CA) and band density was expressed in integrated density units relative to muscle weight.

### Statistical analysis

Statistical analyses were performed using SPSS (version 14.0, SPSS Inc., Chicago, IL). Whole blood, serum, performance, and body composition variables were analyzed using 2 × 3 (group × testing session) analysis of variance (ANOVA) with repeated measures univariate tests. MHC protein, FP and EP_3 _receptor protein levels were analyzed using separate 2 × 2 (group × testing session) ANOVA with repeated measures. Additionally, in the case of significant main effect for group, one-way ANOVAs with repeated measures on testing sessions was performed for each group to assess any differences between tests. Independent t-tests were used to analyze the changes in MHC mRNA expression after 50 days of supplementation. In addition to raw score analysis, delta score analysis (i.e. day 0 values subtracted from day 25 and/or 50) was performed for variables that exhibited extraneous variation between groups on day 0. As mentioned with raw scores, a 2 × 3 (group × testing session) analysis of variance (ANOVA) with repeated measures univariate tests was used to analyze delta scores for body composition, performance variables, and hormone concentrations whereas a 2 × 2 repeated measures ANOVA was used to analyze all intramuscular delta scores. In circumstances where equal variances within groups could not be assumed, the Hunyhs-Feldt epsilon correction factor was used to adjust within group F-ratios. In circumstances where statistical trends appeared to exist (i.e., *P *= 0.05 to 0.10), effect sizes were also reported as partial Eta squared (η_p_^2^). Partial Eta squared effect sizes were determined to be weak (η_p_^2 ^≤ 0.01), medium (η_p_^2 ^= 0.06), strong = (η_p_^2 ^= 0.14) as previously described [38]. Significance for all statistical analyses was determined using an alpha level of 0.05. It should be noted that an *a priori *power analysis of the design indicated that an n-size of 15 participants per treatment would yield a high power (> 0.8) for criterion variable delta values of 0.75 to 1.25. It should also be noted that *post hoc *outlier analysis using box plots was performed in circumstances where there were significant group × time interactions to ensure there were no outliers present. All data are reported as means ± standard deviations (SD).

## Results

### Demographics

Baseline demographics were calculated for both groups (Table 1). No adverse side effects for any subject were reported to the research nurse throughout the study as both the training and supplementation regimens were well tolerated. Dietary protein intake increased by 67.9 ± 33.4 g·day^-1 ^in both groups which increased relative protein intake from 1.3 ± 0.4 g·kg·day^-1 ^(Day 0) to 2.0 ± 0.5 g·kg·day^-1 ^(Day 50). As expected, AA intake significantly increased in the AA group at days 25 (AA: 1.08 ± 0.12 vs. PLA: 0.26 ± 0.50 g·day^-1^, *P *< 0.001) and 50 (AA: 1.09 ± 0.12 vs. PLA: 0.24 ± 0.47 g·day^-1^, *P *< 0.001) compared to the PLA group. No significant group × time interactions were found for dietary linoleic or linolenic acid intake values throughout the course of the study, although linoleic acid intake significantly decreased from day 0 to 50 in both groups (day 0 – day 50 = -3.29 ± 6.42 g·day^-1^, *P *= 0.03). In addition, no between-group differences in training volume were found throughout the study (AA: 4,303 ± 1,291 vs. PLA: 4,105 ± 641 kg·kg bodyweight^-1^, *P *= 0.59).

**Table 1:** Body Composition and Strength Changes.

Variable	AA (n = 15)	PLA (n = 16)	Significance
Body Mass (kg)			
Day 0	85.1 ± 11.5	87.1 ± 14.5	Group: 0.70
Day 25	85.6 ± 11.6	87.9 ± 14.4	Time: 0.001
Day 50	86.6 ± 12.2	88.1 ± 13.0	G × T: 0.31
FFM (kg)			
Day 0	59.9 ± 8.2	63.9 ± 8.4	Group: 0.19
Day 25	60.9 ± 8.1	64.8 ± 8.2	Time: 0.001
Day 50	61.1 ± 8.5	64.9 ± 8.0	G × T: 0.71
Fat mass (kg)			
Day 0	15.8 ± 5.6	13.8 ± 7.3	Group: 0.35
Day 25	15.5 ± 5.7	13.5 ± 6.9	Time: 0.24
Day 50	16.3 ± 5.9	13.8 ± 7.2	G × T: 0.22
%BF (%)			
Day 0	19.9 ± 6.1	16.5 ± 6.5	Group: 0.12
Day 25	19.3 ± 6.1	15.9 ± 5.9	Time: 0.02
Day 50	20.0 ± 6.0	16.3 ± 6.2	G × T: 0.43
Bench Press 1RM (kg)			
Day 0	103.0 ± 24.9	113.5 ± 24.6	Group: 0.26
Day 25	107.0 ± 23.4	118.5 ± 23.0	Time: < 0.001
Day 50	114.4 ± 25.8	121.5 ± 21.5	G × T: 0.20
Leg press 1RM (kg)			
Day 0	370.8 ± 113.5	413.4 ± 80.4	Group: 0.26
Day 25	379.2 ± 109.5	416.8 ± 77.2	Time: < 0.001
Day 50	395.8 ± 123.8	436.1 ± 78.9	G × T: 0.8

Values are expressed as means ± SD.

AA: Arachidonic acid, PLA: placebo, %BF: body fat percentage, 1RM: one-repetition maximum.

### Body composition and performance

Table 1 presents body composition and strength results for the PLA and AA groups. No significant group × time interaction changes were observed among groups in body mass (*P *= 0.31), fat free mass (*P *= 0.71), fat mass (*P *= 0.22), percent body fat (*P *= 0.43), 1RM bench press (*P *= 0.20), or 1RM leg press (*P *= 0.84).

**Figure 1:**
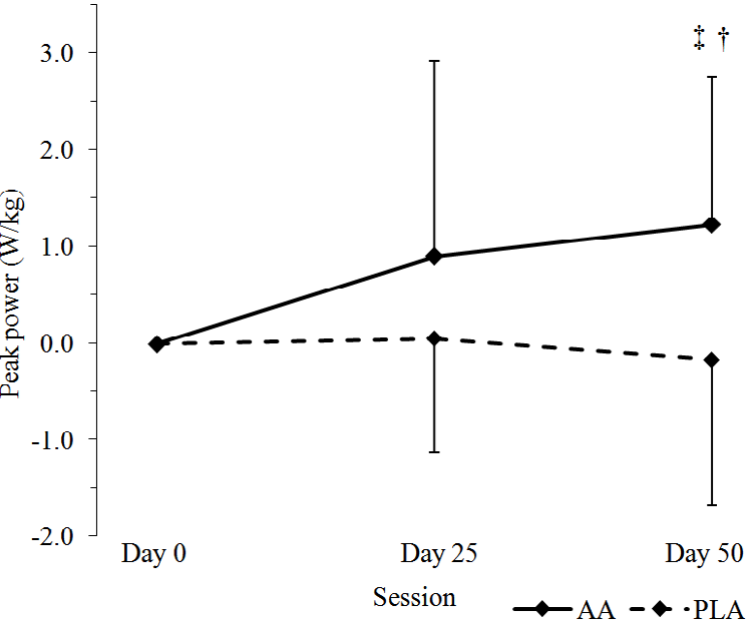
Delta changes in Wingate peak power normalized to body mass over the 50 d supplement intervention. All values are expressed as means ± SD. ^‡^Significantly different from baseline, p < 0.05. ^†^Significant group × time interaction between AA and PLA groups, p < 0.05. AA: Arachidonic acid (n = 15); PLA: placebo (n = 16).

Table 2 presents results of the anaerobic capacity test. A significant group × time interaction for relative Wingate peak power was observed among groups (*P *= 0.02) with gains in peak power being significantly greater in the AA group (0.3 ± 1.2 W·kg^-1^) vs. PLA (0.2 ± 0.7 W·kg^-1^, Figure 1). Using repeated measures ANOVA with delta scores, AA experienced significantly greater increases in comparison to the PLA group at day 50 (*P *< 0.05). Statistical trends were seen in Wingate total work (AA: 1,292 ± 1,206 vs. PLA: 510 ± 1,249 J, *P *= 0.09, η_p_^2 ^= 0.052), favoring the AA group.

**Table 2:** Anaerobic Performance Changes.

Variable	AA (n = 15)	PLA (n = 16)	Significance
Wingate Mean Power (W·kg^-1^)			
Day 0	8.3 ± 1.2	8.9 ± 0.7	Group: 0.17
Day 25	8.5 ± 1.1	8.9 ± 0.8	Time: 0.35
Day 50	8.6 ± 1.2	9.1 ± 0.7	G × T: 0.08
Wingate Peak Power (W·kg^-1^)			
Day 0	16.9 ± 3.1	18.3 ± 2.6	Group: 0.50
Day 25	17.9 ± 2.6^b^	18.3 ± 2.8	Time: 0.11
Day 50	18.1 ± 2.6^b^	18.1 ± 2.8	G × T: 0.02
Wingate Total Work (Joules)^a^			
Day 0	21,102 ± 3,620	22,388 ± 4,222	Group: 0.18
Day 25	21,658 ± 3,307	23,340 ± 3,909	Time: 0.001
Day 50	22,394 ± 4,096	23,899 ± 3,493	G × T: 0.052

Values are expressed as means ± SD.

AA: Arachidonic acid, PLA: placebo.

^a^Significant main effect over time, p < 0.01.

^b^Significant within-group difference from baseline values, p < 0.05.

### General clinical blood analyses

There was no significant group × time interactions for red blood cell, white blood cell count, or white blood cell differentials over the course of the study. Furthermore, no significant group × time interactions existed for serum triglycerides, total cholesterol, glucose, hepatic enzymes (i.e., GGT, AST, ALT), hepatic proteins (i.e., total protein, albumin, bilirubin), electrolytes (i.e., sodium, potassium, chloride, calcium), crude markers of kidney integrity (i.e., ALP, BUN, creatinine, uric acid), or crude markers of muscle damage (i.e., CK, LDH) throughout the duration of the study.

### Anabolic and catabolic hormones/cytokines

**Figure 2:**
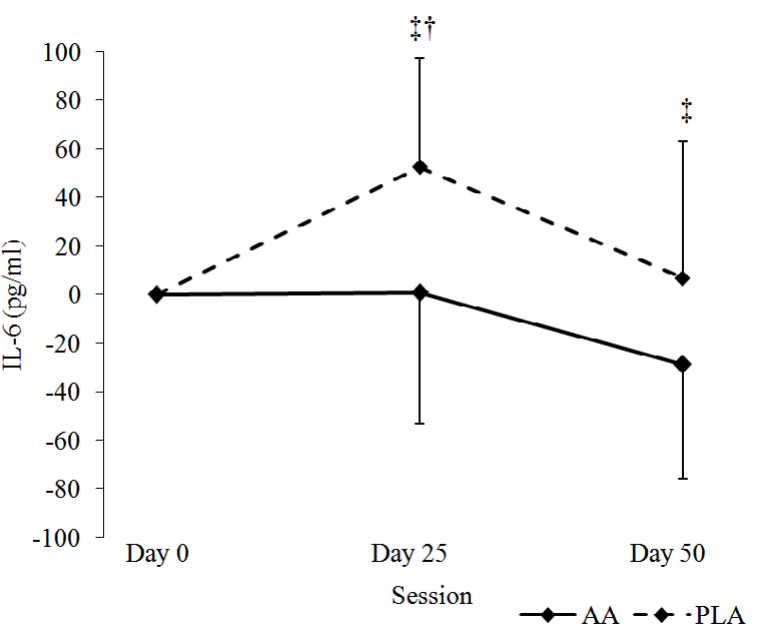
Delta changes in IL-6 levels over the 50 d supplement intervention. All values are expressed as means ± SD. ^‡^Significantly different from baseline, p < 0.05. ^†^Significant group × time interaction between AA and PLA groups, p < 0.05. AA: Arachidonic acid (n = 15); PLA: placebo (n = 16).

No significant group × time effect interactions were observed tTEST (*P *= 0.89), fTEST (*P *= 0.51), and CORT (*P *= 0.81) (Table 3). A statistical trend was found for serum changes in PGE_2 _(AA: 98.5 ± 217 vs. PLA: -73.8 ± 273 pg·ml^-1^, *P *= 0.06, η_p_^2 ^= 0.06). IL-6 levels experienced a significant group × time interaction (*P *= 0.04) among groups with subsequent post-hoc analyses revealing that IL-6 was significantly lower at day 25 of the study. One-way ANOVA of IL-6 delta values at day 25 revealed significantly greater increases in PLA when compared to AA group (AA: 0.8 ± 13.5 pg·ml^-1 ^vs. PLA: 52.5 ± 1.6 pg·ml^-1^, *P *= 0.01; Figure 2).

**Table 3:** Hormone/Cytokine Concentrations.

Variable	AA (n = 15)	PLA (n = 16)	Significance
PGF_2α _(pg·ml^-1^)			
Day 0	164.6 ± 104.4	183.6 ± 96.0	Group: 0.89
Day 25	221.5 ± 153.2	281.5 ± 248.2	Time: 0.06
Day 50	209.7 ± 226.3	150.0 ± 104.5	G × T: 0.14
PGE_2 _(pg·ml^-1^)			
Day 0	224.3 ± 145.7	293.6 ± 256.7	Group: 0.97
Day 25	288.5 ± 191.2	314.8 ± 236.1	Time: 0.65
Day 50	322.8 ± 281.2	219.9 ± 163.6	G × T: 0.06
tTEST (ng·ml^-1^)			
Day 0	21.4 ± 24.8	14.0 ± 12.1	Group: 0.25
Day 25	18.7 ± 22.4	12.2 ± 7.1	Time: 0.24
Day 50	18.7 ± 18.3	12.2 ± 7.7	G × T: 0.83
fTEST (pg·ml^-1^)			
Day 0	25.3 ± 14.7	24.9 ± 19.6	Group: 0.82
Day 25	23.1 ± 12.1	20.0 ± 12.1	Time: 0.03
Day 50	22.3 ± 13.6	22.3 ± 15.0	G × T: 0.87
CORT (μg·dl^-1^)			
Day 0	34.0 ± 24.1	26.2 ± 10.6	Group: 0.44
Day 25	28.2 ± 20.0	23.1 ± 7.8	Time: 0.06
Day 50	36.8 ± 22.9	34.2 ± 35.8	G × T: 0.57
IL-6 (pg·ml^-1^)			
Day 0	137.2 ± 69.6	120.1 ± 92.1	Group: 0.63
Day 25	138.0 ± 83.1^b^	172.6 ± 90.5^a^	Time: 0.001
Day 50	108.4 ± 48.6^a^	127.0 ± 56.8	G × T: 0.03

Values are expressed as means ± SD.

AA: Arachidonic acid, PLA: placebo, PGF_2α_: prostaglandin F_2α_, PGE_2_: prostaglandin E_2_, tTEST: total testosterone, fTEST: free testosterone, CORT: cortisol, IL-6: interleukin-6.

^a^Significant within-group difference from baseline values, p < 0.05.

^b^Significant group × time (G × T) interaction, p < 0.05.

### Intramuscular gene and protein expression

Table 4 presents muscle protein content, MHC fiber protein content, MHC mRNA expression, and FP and EP_3 _receptor levels observed for each group in response to training. A significant group × time interaction (*P *= 0.02) in myofibrillar protein levels was found among groups. Post-hoc analysis revealed that the PLA group (1.6 ± 0.5 μg·g^-1^) demonstrated significantly greater total muscle protein levels at day 50 in comparison to AA (0.5 ± 0.1 μg·g^-1^). No significant group × time interactions were found in FP and EP_3 _receptor density values. Basal MHC gene expression was not affected by AA supplementation as no significant group × time interactions were observed concerning changes in Type I, IIa, and IIx MHC protein or mRNA levels. A significant main effect over time (*P *< 0.01) for changes in MHC IIa protein levels were found in both groups indicating that training primarily promoted gains in MHC IIa fiber protein content. However, no statistical differences were observed between groups.

**Table 4:** Intramuscular Adaptations and MHC mRNA Expression

Marker	AA (n = 15)	PLA (n = 16)	Significance
Total protein (μg·g^-1^)			Group: 0.92
Day 0	7.5 ± 4.5	6.6 ± 3.8	Time: 0.28
Day 50	7.0 ± 4.5	8.2 ± 4.3^a,b^	G × T: 0.05
FP receptor (INT·mm^2^·mg^-1^)			Group: 0.63
Day 0	1,929 ± 1,395	2,253 ± 1,309	Time: 0.13
Day 50	2,425 ± 1,746	2,518 ± 992	G × T: 0.64
EP_3 _receptor (INT·mm^2^·mg^-1^)			Group: 0.55
Day 0	5,990 ± 3,563	6,854 ± 3,287	Time: 0.11
Day 50	7,149 ± 3,383	7,529 ± 2,791	G × T: 0.66
MHC I protein (ng·ml^-1^)			Group: 0.78
Day 0	187.8 ± 180.7	172.8 ± 123.0	Time: 0.64
Day 50	176.3 ± 104.4	164.3 ± 126.6	G × T: 0.96
MHC IIa protein (ng·ml^-1^)			Group: 0.84
Day 0	184.4 ± 217.1	156.9 ± 84.5	Time: 0.009
Day 50	305.0 ± 366.3	295.8 ± 328.5	G × T: 0.84
MHC IIx protein (ng·ml^-1^)			Group: 0.55
Day 0	128.7 ± 79.1	133.6 ± 83.5	Time: 0.23
Day 50	103.9 ± 68.2	131.9 ± 81.7	G × T: 0.29
MHC I mRNA			Group: 0.45
Day 0	0.69 ± 0.16	0.68 ± 0.14	Time: 0.70
Day 50	0.64 ± 0.07	0.71 ± 0.15	G × T: 0.14
MHC IIa mRNA			Group: 0.32
Day 0	0.66 ± 0.12	0.66 ± 0.14	Time: 0.55
Day 50	0.63 ± 0.07	0.71 ± 0.13	G × T: 0.08
MHC IIx mRNA			Group: 0.42
Day 0	0.66 ± 0.14	0.65 ± 0.15	Time: 0.98
Day 50	0.62 ± 0.07	0.69 ± 0.14	G × T: 0.10

Values are expressed as means ± SD.

AA: Arachidonic acid, PLA: placebo, INT: integrated density units, MHC: myosin heavy chain.

^a^Significant within-group difference from baseline values, p < 0.05.

^b^Significant group × time (G × T) interaction, p < 0.05

## Discussion

Prior preliminary research has suggested that prostanoid signaling may mitigate post-exercise inflammation and/or muscle protein synthesis [18,39]. For this reason, AA supplementation has been marketed as a dietary supplement designed to promote muscle hypertrophy in resistance trained athletes. To date, we are not aware of any study that has been conducted to assess this theoretical role of AA supplementation during resistance-training. Therefore, the purpose of this study was to determine whether AA supplementation during training affects body composition, circulating hormones, intracellular markers of muscle hypertrophy, and/or exercise performance in experienced resistance-trained males. The major findings from this study were that AA supplementation significantly increased anaerobic peak power by 8.5% at day 50 (*P *< 0.05) and attenuated the increases in circulating IL-6 levels that were seen in the PLA group (AA: 138.0 ± 83.1 pg·ml^-1^, PLA: 172.6 ± 90.5 pg·ml^-1^, *P *< 0.05) on day 25 of the study. Statistical trends were also found for PGE_2 _increases (98.5 ± 217 vs. PLA: -73.8 ± 273 pg·ml^-1^, *P *= 0.06) in the AA group. These findings suggest that AA supplementation may increase prostaglandin levels in the blood and provide a potential ergogenic value for athletes engaged in high-intensity exercise. Additionally, that subjects engaged in intense training may be able to tolerate training with less inflammation as indicated by lower IL-6 levels. However, no significant differences were seen between groups in changes in body composition, circulating anabolic hormones, and/or intramuscular markers of muscle hypertrophy. These findings do not support claims that AA supplementation during resistance training stimulates muscle hypertrophy leading to greater gains in strength and/or muscle mass.

### Changes in body composition and performance variables

Gains in strength and fat free mass for all subjects were similar to results previously reported using this linear periodization training program. However, AA supplementation did not induce statistically greater gains in strength or fat free mass during training when compared to the PLA group. Although more research is needed, these findings do not support claims that AA supplementation during resistance-training promotes lean tissue accretion. Interestingly, AA supplementation significantly increased relative peak power during the Wingate test (*P *= 0.02). Post-hoc analysis revealed that relative peak power was greater in the AA group after 50 days when compared to the PLA group. Statistical trends were also observed in mean power (*P *= 0.08) and total work (*P *= 0.052) performed during the 30-second sprint test. The Wingate anaerobic capacity test is considered to be a valid and reliable assessment of anaerobic power and capacity. Therefore, these findings suggest that AA may enhance anaerobic capacity. In support of this finding, recent literature suggests that 10 days of soybean-derived phosphatidylserine supplementation can significantly increase the time-to-fatigue and total work performed in active males during an 85% VO_2_max cycling bout [40]. Phosphatidylserine is a soy bean-derived phospholipid that contains AA [41]. Kingsley *et al. *(2006) hypothesized that phosphatidylserine supplementation may increase exercise capacity by delaying fatigue and sustaining intracellular calcium levels through an increase in the density of Ca^2+ ^ATPase pumps in the sarcoplasmic reticulum of skeletal muscle [40]. Collectively, these findings provide preliminary framework that AA may have been responsible for the significant increase in Wingate peak power. However, neither our lab nor Kingsley's group quantified acute changes in intramuscular calcium during exercise. Hence, further research is needed to examine the role of AA on intramuscular calcium and anaerobic exercise capacity before these conclusions can be drawn.

### Hormonal adaptations

The analysis of anabolic and catabolic hormonal responses to training has been used as general indicators of training status. For example, reductions in total and/or free testosterone and/or excessive increases in cortisol during training have been related to overtraining [42]. Furthermore, analysis of the ratio of testosterone: cortisol has been used as a general marker of anabolic: catabolic status in trained athletes [42]. Theoretically, improving the ratio of testosterone to cortisol may enhance the hormonal environment for recovery and/or promote muscle hypertrophy during training. Results from the present study indicate that AA supplementation did not significantly affect resting levels of tTEST, fTEST, or CORT. These findings indicate that AA had no effect on the anabolic and/or catabolic status during training in experienced resistance-trained individuals.

While AA supplementation appeared to not affect PGF_2α _levels (*P *= 0.14), AA supplementation tended to increase circulating levels PGE_2 _(AA: 98.5 ± 217 vs. PLA: -73.8 ± 273 pg·ml^-1^, *P *= 0.06). While not statistically significant, this represented a 44% increase in PGE_2 _levels in the AA group over the 50 d supplementation period compared to a 25% decrease in the PLA group. These findings tend to support contentions that AA supplementation may influence prostaglandin levels. The decrease in PGE_2 _in the PLA group may be attributed to the restriction of AA containing foods in the diet. As mentioned previously, Kelly *et al. *[19] demonstrated that supplementing young, healthy men with AA increased the in vitro leukocytic secretion of PGE_2 _upon stimulation with influenza microtiters. However, no previous study to our knowledge has demonstrated that AA supplementation increases resting serum levels of PGE_2 _and/or PGF_2α_. Additional research should examine the acute and chronic effects of AA supplementation on PGE_2 _and PGF_2α _levels in relation to exercise and inflammation.

Interleukin-6 is a cytokine that increases in response to acute inflammation [42]. Intense exercise has been reported to increase IL-6 secretion from the muscle by as much as 100-fold in humans [43]. Consequently, IL-6 is often used as an indicator of muscle inflammation status in athletes. Past research also contends that IL-6 acts as a pro-inflammatory cytokine thereby initiating intramuscular proteolytic activity by up-regulating lysosomal, cathepsin, and ubiquitin pathways [44-48]. In fact, one of the initial theories related to the potential ergogenic value of AA for athletes was that dietary supplementation of AA during training would promote greater localized inflammation thereby stimulating subsequent intramuscular protein synthesis during resting/repair phases between workouts. Nonetheless, present findings do not support this hypothesis. Rather, results suggest that AA supplementation during training may reduce chronic levels of IL-6 thereby reducing associated inflammation that may occur during high volume training. Theoretically, this may allow athletes to tolerate high levels of training to a better degree. However, more research is needed to examine the acute and long-term effects that AA has on intramuscular muscle proteolysis following resistance training before conclusions can be drawn.

### Intramuscular adaptations

Prolonged resistance training has been reported to increase the mRNA and protein expression of the MHC type IIa isoform while decreasing type IIx and minimally altering type I isoform expression patterns [43]. Furthermore, the aforementioned protein expression pattern of these MHC isoforms in skeletal muscle has been highly correlated with muscular hypertrophy [42]. Since AA has been purported to promote muscle hypertrophy, we used these markers to help determine whether AA influenced cellular adaptations to training. Previous research has shown that AA and PGF_2α _both up-regulate phosphoinositol-3 kinase (PI-3K)-p70^S6k ^signaling in vascular smooth muscle cells. This intracellular pathway in skeletal muscle is known to initiate mRNA translation and is also highly correlated with skeletal muscle hypertrophy [49-52]. Nonetheless, results of this study indicate that changes in MHC protein expression were similar in both groups. Therefore, AA supplementation during training had no effect on MHC protein expression.

Myofibrillar protein content is also one of several markers of muscle hypertrophy. Theoretically, if AA promoted muscle hypertrophy, protein content of muscle fibers would increase as has been previously shown in muscle hypertrophy research. Yet, there was no evidence that AA promoted increases in a number of markers of muscle hypertrophy. In fact, myofibrillar protein levels were actually significantly increased in the PLA group while remaining unchanged in the AA group. While past research suggests that PGE_2 _induces lysosomal proteolysis in cultured myotubes [53], it is currently not known if AA mechanistically alters myofibrillar protein levels. Present findings indicate that AA supplementation had no influence on muscle hypertrophy during training as indicated by lack of effect on myofibrillar protein content, MHC protein levels, and fat free mass.

The FP and EP_3 _receptors are ubiquitously expressed throughout the body and exhibit pleiotropic metabolic responses when stimulated. Of particular interest, cardiomyocyte hypertrophy through activation of the ERK2 pathway has been shown to occur through the FP receptor [49]. Similarly, EP_3 _receptor signaling mitigates decrements in cyclic AMP production [54] which promotes the signaling of the mammalian target of rapamycin (mTOR) as well as subsequent assembly of the eukaryotic initiation factor-4G (eIF-4G) [55]. Due to the fact that both of these responses downstream from these receptors are integral to muscular hypertrophy, we chose to examine if AA supplementation increased the expression of the FP and EP_3 _receptors. Contrary to our initial hypothesis, however, our findings reveal that AA did not affect chronic FP and/or EP_3 _receptor densities. Regardless, future research should examine if the abovementioned pathways are acutely increased after exercise.

## Conclusion

A potential limitation to this study was that the PLA group ingested a corn oil supplement which is a source of linoleic acid substrate (22.9 mg·g oil^-1^) that can be converted into arachidonic acid through a multi-catalytic process involving the Δ6-desaturase, elongase, and Δ5-desaturase enzymes, respectively [56]. However, it should be noted that dietary analysis revealed no significant changes between groups in linoleic acid intake throughout the duration of the study. Another interesting finding was the significant reduction in IL-6 levels within the AA group at day 25 and not day 50. While past research has examined dietary-induced changes in intracellular fatty acid stores and cytokine production via peripheral blood mononuclear cell stimulation *in vitro *[57], we measured IL-6 and prostanoid levels in duplicate from serum samples. Therefore, future research should be employed using analytical techniques similar to Bell *et al. *[57] assessing the cellular production of prostanoids and cytokines after ingesting AA over prolonged periods. Beyond these potential limitations, our results suggest that prolonged AA supplementation may increase cycling anaerobic capacity while reducing circulating IL-6 levels in resistance-trained males. Moreover, that 1.0 g·day^-1 ^of AA administration over a 50 days period significantly increased dietary AA intake and appears to be well-tolerated and exerts no adverse side effects in young, resistance-trained males. These findings provide preliminary evidence that AA supplementation may have some benefit for individuals engaged in intense resistance-training. However, although some potentially beneficial trends were observed, AA supplementation had no statistically significant effects on strength, muscle mass, hormonal markers of anabolism/catabolism, or intracellular markers of muscle hypertrophy. Whether training status, dosage, supplement timing, length of supplementation, sample size, and/or the type of training may yield more positive results remains to be examined with additional research. Additionally, whether some athletes may respond better to AA supplementation than others should be explored.

## Competing interests

Molecular Nutrition (Jupiter, FL) provided funding for this project through an unrestricted research grant to Baylor University. All researchers involved independently collected, analyzed, and interpreted the results from this study and have no financial interests concerning the outcome of this investigation.

## Authors' contributions

MDR: primary author, oversaw all aspects of study including recruitment, data/specimen analysis, and manuscript preparation

MI: study co-collaborator, statistical analysis, subject recruitment

CMK: manuscript preparation, data collection, specimen analysis

LWT: specimen and statistical analysis

BC: specimen and statistical analysis

CDW: data collection, statistical analysis

TH: data collection and specimen analysis

MC: data collection and specimen analysis

CR: coordinator of testing facilities and assisted in data collection

MG: secondary author

RW: served as medical director for study

JJ: data collection and dietary analysis

DW: laboratory director of specimen analytical lab, obtained muscle sample, and directed specimen analysis

RBK: principal investigator of the study who obtained grant funds for project, designed study, supervised data collection and analysis, supervised statistical analyses, and co-authored paper
